# Using consecutive Rapid Participatory Appraisal studies to assess, facilitate and evaluate health and social change in community settings

**DOI:** 10.1186/1471-2458-6-68

**Published:** 2006-03-15

**Authors:** Colin S Brown, Simon Lloyd, Scott A Murray

**Affiliations:** 1 Research Fellow, Primary Palliative Care Research Group, Division of Community Health Sciences: General Practice Section, University of Edinburgh, EH8 9DX, UK; 2 Research Fellow, Primary Palliative Care Research Group, Division of Community Health Sciences: General Practice Section, University of Edinburgh, EH8 9DX, UK; 3 Clinical Reader, Primary Palliative Care Research Group, Division of Community Health Sciences: General Practice Section, University of Edinburgh, EH8 9DX, UK

## Abstract

**Background:**

To investigate how a relatively socio-economically deprived community's needs have changed over time, assess which recommendations from an earlier assessment were implemented and sustained, and consider whether serial Rapid Participatory Appraisal is an effective health research tool that can promote community development and has utility in assessing longitudinal change.

**Methods:**

Rapid Participatory Appraisal involves communities in identifying and challenging their own health-related needs. Information on ten health and social aspects was collated from existing documentation, neighbourhood observations, and interviews with a range of residents and key informants, providing a composite picture of the community's structure, needs and services.

**Results:**

The perceived needs after 10 years encompassed a wide construct of health, principally the living environment, housing, and lack of finance. Most identified upstream determinants of health rather than specific medical conditions as primary concerns. After the initial Rapid Participatory Appraisal many interviewees took the recommendations forward, working to promote a healthier environment and advocate for local resources. Interventions requiring support from outwith the community were largely not sustained.

**Conclusion:**

Rapid Participatory Appraisal proved valuable in assessing long-term change. The community's continuing needs were identified, but they could not facilitate and sustain change without the strategic support of key regional and national agencies. Many repeatedly voiced concerns lay outwith local control: local needs assessment must be supported at higher levels to be effective.

## Background

In 1992, we assessed the health and social needs of over 1100 residents of a deprived housing estate in Edinburgh, using Rapid Participatory Appraisal [1]. Ten years later we repeated this assessment to investigate how the needs of a defined, local community had changed over time, establish what recommendations from the initial assessment had been implemented and sustained, and consider factors which may have influenced this.

The method provides a unique means of involving the community in identifying its own health-related needs, important both as a democratic goal and as a potentially useful means of achieving improvements in health [2]. It can provide timely, relevant information, placing such needs within the community's social, economic and cultural context [3]. As an action research method it can facilitate change more than traditional methods of health needs assessment, and a dynamic process of community health promotion can be achieved through both intervention and evaluation [4].

Given the renewed interest in community-based primary health care, through this we wished to assess if Rapid Participatory Appraisal is a simple, effective health research tool that can promote community development, and subsequently be used to evaluate the process and outcomes by having utility in assessing longitudinal change. A number of community needs assessments have been conducted within different population groups such as the homeless [5], those affected by HIV [6], at a neighbourhood level [7], and within primary care settings [8]. The difficulty in evaluating the effects of community development initiatives [9] has resulted in only a few examples concerning long term outcomes; several initiatives have exemplified how a dynamic process of community health promotion can be achieved through both intervention and evaluation [10-12]. If proved effective for Rapid Participatory Appraisal, this would mean that it may be a valuable method to ascertain baseline measures of health and social needs, could be adapted to local situations, and used to monitor various indicators. Furthermore, as it also promotes human development, it may be a key resource in providing sustainable social and economic progress, as well as facilitating health improvements.

## Methods

### Process of gathering information

**Figure 1:**
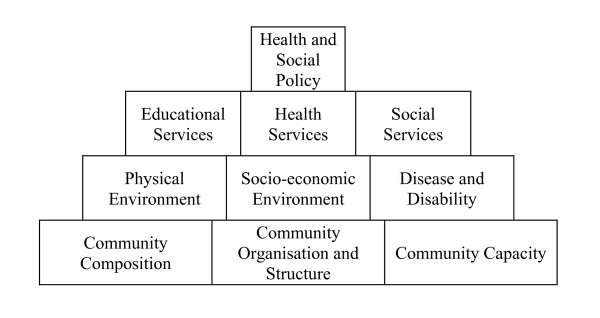
**Information pyramid for Rapid Participatory Appraisal**. A ten-stage composite pyramid diagram detailing the community structure, physical and social environment, health profile, and local and national levels of service provision.

The primary aims of Rapid Participatory Appraisal are to gain insight into a community's own perspective of its main needs; to translate these findings into action; and to establish an ongoing relationship between service providers and local communities. It is "rapid" in that the exercise can be completed in a relatively short time frame. Information on ten aspects of the community was collated, providing a composite picture of the community's structure, needs and role within existing service provision. These are brought together to form an information pyramid (Figure 1). The World Health Organisation interview training material was useful in the devising the semi-structured interview that developed each aspect [see Additional file 1] [2].

The bottom layer defines the composition of the community, how it is organised, and its capacity to act to redress inequalities. The second layer covers the socioecological factors that influence health. The next layer covers data on the existence, coverage, accessibility, and acceptability of services, which allows the effectiveness of present services to be evaluated and identifies what needs to be changed. The final layer is concerned with national, regional, and local policies that indicate whether the political leadership is committed to community participation in health.

We gathered data from three sources: existing written records about the neighbourhood, interviews with a range of informants, and observations made in the neighbourhood or in the homes of interviewees. In this way, findings could be checked by comparison with two other sources of information, affording a more reliable composite picture. The scientific rigour and validity of the approach depends on the concept of triangulation, with data collection from one source being validated or rejected by checking it with data from at least two other sources or methods of collection. Through cross checking findings apparent differences may resolve themselves, and a coherent interpretation can be constructed. Furthermore Rapid Participatory Appraisal was included as part of a complementary four-stage study that included routinely held Health Service and Census data, locally held General Practice information, and a postal survey. This composite model was thought to be most informative way of identify need. The interviewees were purposively selected to represent a broad knowledge of the community, and were drawn from a range of professional, community and campaigning backgrounds. Twelve of the 27 interviewees were residents, selected to represent various age groups, social situations, and health issues. Only 4 of these interviewees had been interviewed in 1992 (Table 1).

### Local context: strategy for change

Dumbiedykes is a housing estate of over 1100 residents in central Edinburgh with high levels of poverty and unemployment, lack of social cohesion, and widespread morbidity. It is served predominantly by one local general practice whose health professionals took an active interest in the health and social needs of the residents. At the initial rapid appraisal, few interviewees recognised major health problems considered to be national priorities as areas of most concern. Rather, they identified problems with their living conditions, housing and economic situation. Many residents considered that the local environment adversely affected their health and felt unable to improve it. They felt that there was inadequate provision of local, relevant services. At this neighbourhood level, public health and primary care services did not appear to address such issues.

The local residents and key local professionals and volunteers who had been interviewed were then invited to form a health forum in Dumbiedykes to advocate for the suggestions raised by the rapid appraisal process, such as a bus into the estate, provision of play areas, housing improvements and more accessible social services. They have continued to regularly meet to plan activities and advocate for resources for the area, identifying and addressing key local issues as they arose.

After the second Rapid Participatory Appraisal we compared the 2002 findings with those from 1992, examining for evidence that any of the previous recommendations were implemented, and ascertaining the perceived success or failure of any intervention.

## Results

### Current health and social situation in Dumbiedykes

In 2002, the residents perceived that after 10 years the main issues and needs still encompassed a wide construct of health, principally the living environment, housing, and lack of financial resources (Table 2). Factors identified as influencing health again included unemployment, damp, diet, smoking, poverty, and the estate's appearance. Once more, few identified diseases considered national priorities, such as cardiovascular and respiratory disease, as primary concerns, most identifying upstream determinants of health (the political, economic, environmental and socialfactors that shape societies and the opportunities to be healthy within them) [13] rather than specific medical conditions. Alcohol and substance abuse remained a noticeable problem, particularly among the youth.

Lack of maintenance of the council housing was the most heartfelt unmet need, and was frequently voiced when health was discussed. This was partly due to prolonged delays in repairs while "stock transfer", whereby the council transfers ownership to a Housing Association, was being considered. This delay and division unfortunately decreased social cohesion, and the proposed stock transfer was eventually abandoned due to national policy change.

### Effectiveness of interventions implemented since the initial needs assessment

The bus still runs, and the central heating has been upgraded. The introduction of an affordable food co-operative has raised awareness and increased accessibility to means of healthy eating. The strong desire for a neighbourhood focal point was met after eight years by the construction of a community centre. Through the Health Forum's and local residents' committees' efforts, this council-funded conversion of a disused nursery provides what the residents have wanted: a central area that enables them to meet, plan activities for the youth and the elderly, and expand the food co-operative into a healthy café. It is anticipated to support a diverse range of health and social services, and provide a base for outreach into and encourage more active participation from the wider community. The Health Forum has also successfully promoted a healthier environment by decreasing litter and introducing safe play areas, and advocated for increased local resources.

Despite these initiatives, interventions requiring support from outwith the community were largely not sustained. Many problems remain, with key issues identified being the continued lack of investment in housing, and, despite community-led initiatives such as writing, education and drama groups, a real need for facilities for the youth. This may be partially remedied by the opening of a new local and affordable sports complex, with Health Forum backing. In Table 3 we list the recommendations from the first study, and assess whether they have been implemented and sustained.

## Discussion

### Lessons learnt

Since the early 1990s there has been a resurgence of interest in the role of place in shaping people's health experiences [14]. Geographic definitions of communities are meaningful to citizens, and institutions that identify local needs have more credibility when attempting to engage in local health promotion [15]. Our findings concur: not only do people feel strongly about the needs of their community, but may feel restricted by the inherent limitations of their neighbourhood's design, and by political and economic influence. Indeed, many residents in this location consider a great number of external considerations and "life circumstances" to be the major determinants of health [16]. When community-based health care providers are determining service provision, such considerations must be taken into account. Indeed, this case study illustrates that Rapid Participatory Appraisal is an effective developmental method which can be utilised as a catalyst through which health and social work professionals can gain a community orientation and bring about changes. We consider that this is a timely illustration of what can be done when, after many years of rhetoric, the practice of primary care, public health and social care are coming together in the UK in organisations such as a Local Health Partnerships. Rapid Participatory Appraisal also allows for a crosscutting approach to understand and consider how local people feel that their neighbourhood can be a healthier and better place in which to live. Nuances in community health beliefs, structure, and access to resources can be elucidated, and will affect how best to assess current levels of health and social need, and which approach is most appropriate to implement change.

### Limitations of rapid appraisal

Despite choosing a broad range of individuals to represent the community, the risk of not representing some members is present, although the triangulation of data from other sources lessens this. A researcher bias may exist because of professional training, ethnicity, sex and theoretical perspectives. Assessing 'needs' is difficult, as most variables identify 'demand', which may in turn alter with supply of services [17]. Our findings are neighbourhood specific, with distinctive factors including the unique environs with the construction of the new Scottish Parliament, and upmarket housing within 100 metres. Nevertheless, the study describes processes and highlights issues that are relevant to similar investigations into social and health needs worldwide, as this method is applied best to a population that can be considered as a community in some sense of the word. As such it has utility on both a local and national context. We have previously critiqued the major limitations of rapid appraisal [18].

### Strengths of rapid appraisal

The aim of the initial Rapid Participatory Appraisal was to assess the health and social needs of people living in a well defined deprived area, rather than to a bring a community orientation to the primary care services. An early finding from the interviews was a lack of social cohesion – this was identified and prioritised by informants, and interventions of a community development nature were started. These interventions were quite distinct from improvements to primary care services. However the willingness of local health professionals to participate in community development was particularly welcomed and was perceived as being very supportive to local leaders. Though impossible to solely attribute the health improvements in the community to these initiatives, what has been achieved is a growing sense of community, as local residents operate a food co-operative and café, and succeed in advocating for housing and transport improvements. Most residents can identify key community members who are active for their neighbourhood. Research suggests that community empowerment and associated increased social capital can effect improvements in health, and this work may have helped foster this [19]. This increased social interaction has been reported as affecting issues ranging from "promotion of successful youth development to the encouragement of political participation".

Rapid appraisal can be carried out with limited resources, and furnish clinicians and planners with rich insights into local communities; for those in resource poor settings it is therefore a worthwhile first step to assess baseline health and social measurements. Moreover, as an action research method it facilitated changes even before the Health Forum started to meet; this ability to create capacity from within the community is one of its greatest strengths. By promoting intersectoral communication and co-operation using an approach from outwith the traditional medical model, Rapid Participatory Appraisal can help community planners focus on development outcomes rather than simply assess need. In doing so, it may help form long-term partnerships between civil society, Non-Governmental Organisations, and government services.

Longitudinal studies using methods such as rapid appraisal may be necessary to redress the lack of documented long-term outcomes of community development initiatives [9]. Such studies may be necessary to assess whether local community involvement works beyond engagement to confer tangible social and health improvements.

## Conclusion

### Next Steps: local, national and international context

In 1883 the first Minister of Health for Glasgow warned of the danger of divorcing everyday clinical care from population aspects. Pickles [20], Hart [21], and others have practised and advocated for an explicit public health dimension to primary health care. The time is now ripe to practise Community Oriented Primary Care as mandated by the World Health Organisation Alma Ata Agreement, which seeks to bring together the health care of the community and individuals in a single integrated practice that endeavours to identify the community's main health problems and implement solutions to deal with these, while at the same time providing clinical care for individuals [22]. This was originally developed in rural South Africa and Israel, has had considerable success in the USA, and has a growing band of advocates in the Australia and the UK, where public involvement has to date been minimal [23]. Iliffe and Lenihan detail its trails within primary care in the UK, and by reviewing its application in America, suggest that "attention to the factors that promote and impede success, to methods of engaging local communities, and to the development of reliable outcome measures is urgently needed [24]." Community Oriented Primary Care requires a sustained commitment of resources for success, and seems an ideal method for which to facilitate improvements in health and social care [25]. RPA may be a useful mechanism for its initial assessment and implementation.

Our project may have contributed to specific local improvements and, perhaps more importantly, instigated a forum that drives ongoing locally determined actions. For this reason we shall now play a more background role to support and resource both this Health Forum and the local residents' committees. Rapid Participatory Appraisal proved valuable in assessing long-term change, however this cannot occur in isolation: instead they must be viewed within the constructs of social, cultural, environmental and political factors. The community's continuing needs were identified, though it is apparent that communities cannot engage in health promotion, and facilitate and sustain change without the strategic support of key regional and national agencies. In the case of Dumbiedykes, such agencies, although sympathetic to increased local provision, have been driven, due to economic reasons, to cut back on local services which were greatly appreciated, especially by the elderly and the young. Even with strong advocacy from the Health Forum to the local health and social work authorities, local chiropody and nursery provision were closed, to permit economies of scale. This process has mirrors worldwide, and is well known to community planners. The repeatedly voiced concerns lying outwith local control suggest that local needs assessment should be supported at a regional and national level. Only through combined consultation and partnership strengthening can the health and social needs of communities be realised. However local residents and professionals, due to meeting and working together, have a good mutual understanding.

Considering a national model may help the move towards an open, global partnership for development. In the UK, National Health Service policy has encouraged general practitioners, community nurses and public health specialists to assess needs and plan health and social services at a local level, with public participation [26]. This call is currently loudly renewed in a government plan for *Community Health Partnerships *which are being tasked to plan joint futures with primary health care teams, local authorities, public health departments, secondary care institutions, community development leaders and the public [27]. These groups will determine local service provision, and Rapid Participatory Appraisal has the potential to assess and lead to improvements in the quality and acceptability of services in this process at both a national, as well as at an individual community level.

We have found Rapid Participatory Appraisal to have benefits in increasing community engagement, fostering human development and community action, promoting joint local government and health service co-operation, and enabling locally driven service development through identifying ways to provide better service delivery. It would therefore seem timely to promote, utilise and further develop rapid appraisal methods.

## Competing interests

The author(s) declare that they have no competing interests.

## Authors' contributions

CSB designed the 2002 study, undertook the interviews and other data collection, and wrote the paper. Having carried out the 1992 study, SAM helped design the 2002 study, assisted with the analysis, and wrote the paper. SL undertook the interviews with CSB, helped with the analysis, and wrote the paper.

## Pre-publication history

The pre-publication history for this paper can be accessed here:



## Supplementary Material

Additional file 1 Rapid Appraisal Questions. Example of questionnaire used to compile data from key informants and residents Click here for file

## Figures and Tables

**Table 1:** List of informants interviewed in the initial and follow-up rapid appraisals

**2002 rapid appraisal** Manager, Dumbiedykes Information Technology Centre Project co-ordinator, Old Town Community Development Housing department officer, Edinburgh District Council Voluntary worker, St Anne's Community Centre Minister, local Apostolic Church Local general practitioner Local community involvement police sergeant Editor, Dumbiedykes Writers Group Lothian regional counsellor Two local health visitors Chair and former chair, Dumbiedykes Residents Association Shopkeeper, Dumbiedykes Store Project co-ordinator, local youth project Twelve residents were also selected to represent various age groups, social situations, and health issues
**1992 rapid appraisal** Voluntary Worker, St Ann's Community Centre Visiting Sister, St Patrick's Roman Catholic church Home Care Organiser, Social Work Department Project Director, South Side Care Project Dumbiedykes Social Club Convenor Regional Counsellor Project Coordinator, Safer Edinburgh Project District Counsellor Local Community Involvement Police Officer Receptionist, Mackenzie Medical Centre Community Development Worker Old Town Renewal Trust Housing Department Officer, Edinburgh District Council Pharmacist Local District Nurse Head Teacher and Deputy Head Teacher, Local Primary School Volunteer, Women's Royal Voluntary Service Community Psychiatric Nurse, Community Drug Problems Service Shopkeeper, Dumbiedykes Store Project Coordinator, Local Youth Project Local Health Visitor Public Transport Unit, Planning Department, Regional Council Coordinator, Dumbiedykes Children's Centre Recently retired local General Practitioner Group interview – South Side Care Project Board of Directors Group interview – Reminiscence Group Group interview – Dumbiedykes Residents Association Group discussion – teenage girls at youth project Seventeen residents were also selected to represent various age groups, social situations, and health issues

**Table 2:** Key health and social needs identified by informants at the second appraisal

• Environment- Dampness, poor lighting, and unsealed window frames remain key housing concerns. Residents feel 'ashamed' of the condition of their houses, blaming the council for years of neglect. There remains a paucity of affordable local shops. The hills and numerous steps in the estate remain a prominent complaint. Rubbish and dog litter are reported less. Violence was thought no more prominent than elsewhere, although people were still fearful at night.
• Socio-economic factors- The economic outlook has improved little, with many, particularly young families and the elderly, being most in need. Unemployment remains high, though more residents are now owner-occupiers.
• Health and social services- Access to care has worsened: the move of the local city centre hospital to the outskirts was bemoaned and local chiropody and social work nursery provision has been closed. Community based initiatives are well used and appreciated, but awareness of non-local health and social services remains poor.

**Table 3:** Interventions and outcomes of first Dumbiedykes Community Needs Assessment

**Intervention**	**Outcome**
Bus into estate	After being instigated and running for 5 years, the route was altered due to Local Authority cost constraints. It was re-instated with advocacy from the Health Forum.
Provision of multiple play areas	After discussion with the council, fenced off, lawned areas for children were constructed, but were later covered with shrubs rendering them unsuitable as play areas.
Citizens' advice in estate	Ceased due to under use, however notice boards remain to inform people of key issues and ways to get advice.
Local counsellor surgeries in estate	Well used by residents, and counsellor now attends the Health Forum
Change in housing allocation by Council	After the first study revealed that high numbers of people with serious mental illness were allocated to the area, there is now decreased placement of similar tenants.
Medical input into home-help training	Took place for one year. Supervisor remains in contact with Health Forum
Community education sessions	Encouraging response to initiative of health related courses. This led to further development and funding of a well-equipped computer centre, based in an old community room.
Supporting local residents' associations.	Support for associations was appreciated by residents, especially in consulting Council over housing conditions. However little progress was realised due to financial constraints in housing.
Improvements in provision of primary care.	New phone line, ramp, improvement of repeat prescribing system, and increased availability of appointments were implemented and remain in place.
Health Visitor embracing a community development role.	The community has welcomed active involvement by the local practice's health visitors, especially with the food co-operative, and nurse-led initiatives.
Campaign to increase access to local health and social services.	Services such as chiropody and nursery provision, despite local advocacy, have been centralised.
