# Supplementary material for: Using consecutive Rapid Participatory Appraisal studies to assess, facilitate and evaluate health and social change in community settings
Source: BMC Public Health. 2006 Mar 15;6:68. doi: 10.1186/1471-2458-6-68 (PMC1435890; doi:10.1186/1471-2458-6-68)
Supplement: Additional file 1 — Rapid Appraisal Questions. Example of questionnaire used to compile data from key informants and residents [file 1471-2458-6-68-S1.doc]

# Rapid Appraisal Questions

# Community Composition

# *Can you describe the kinds of people who live in the area?*

# Community Organisation and Structure

# *Do you know of what kind of help is available for the residents of Dumbiedykes?*

*Can you think of any other services that would be helpful to people in the area?*

**3. Community Capacity**

*Do you know local people who are good at getting things done?*

*Do you think there is a sense of community identity and/or commitment to this area?*

**4. Physical Environment**

*Are there any particular problems with living in the area?*

*How would you describe the condition of housing in Dumbiedykes?*

*Does transport or access present you with any problems?*

*How safe do you feel in the neighbourhood e.g. walking outside after dark, or being home alone? If not, why?*

*Are you aware of any environmental health problems in the area?*

# Socio-economic Environment

# *Are you aware of a lot of people who find it hard to manage financially?*

*Do you know if violence/crime is an issue?*

**6. Disease and Disability Profile**

*What kinds of things do you think affect the health of people living in Dumbiedykes?*

*What do you think are the worst health problems in the area?*

*Have these changed over the last few years?*

*Do you know if drug abuse is a problem?*

*Has this changed over the last few years?*

*Do you know if alcohol abuse is a problem?*

*Has this changed over the last few years?*

*Are there many people with a physical, learning or mental disability living in this area?*

*Has this changed over the last few years?*

**7. Educational Services**

*Are you aware of these services locally?*

Nurseries

Primary Schools

Secondary Schools

Community Centres

Day/Evening Classes

Youth Club

*How could these be improved?*

*What else would you like in the area?*

**8. Health Services**

*Are you aware of these services locally?*

General Practitioners

Health Visitors

Nurses

Occupational Therapists

Chiropody

Physiotherapists

Dentists

*What is the best thing about the service and what could be better?*

*What do you think of the hospital services?*

*Have you noticed any recent changes in these services?*

*How would you like to see them improved?*

**9. Social Services**

*Which social services are needed by most people in the area?*

*How could they be improved?*

**10. Health and Social Policy**

*Are you aware of government Health and Social Policy? Have any recent changes in policy affected you?*

**11. Miscellaneous**

If you could wave a magic wand what changes would you like to make in the area?
